# Temporal vocal features suggest different call-pattern generating mechanisms in mice and bats

**DOI:** 10.1186/1471-2202-14-99

**Published:** 2013-09-10

**Authors:** Steffen R Hage, Natalja Gavrilov, Ferdinand Salomon, Anna M Stein

**Affiliations:** 1 Animal Physiology, Institute of Neurobiology, University of Tübingen, Auf der Morgenstelle 28, Tübingen, 72076, Germany

**Keywords:** Acoustic communication, Bat, Mammal, Mice, Mouse model, Vocal pattern generation

## Abstract

**Background:**

Mice produce ultrasonic vocalizations in various inter-individual encounters and with high call rates. However, it is so far virtually unknown how these vocal patterns are generated. On the one hand, these vocal patterns could be embedded into the normal respiratory cycle, as happens in bats and other mammals that produce similar call rates and frequencies. On the other, mice could possess distinct vocal pattern generating systems that are capable of modulating the respiratory cycle, which is what happens in non-human and human primates. In the present study, we investigated the temporal call patterns of two different mammalian species, bats and mice, in order to differentiate between these two possibilities for mouse vocalizations. Our primary focus was on comparing the mechanisms for the production of rapid, successive ultrasound calls of comparable frequency ranges in the two species.

**Results:**

We analyzed the temporal call pattern characteristics of mice, and we compared these characteristics to those of ultrasonic echolocation calls produced by horseshoe bats. We measured the distributions of call durations, call intervals, and inter-call intervals in the two species. In the bat, and consistent with previous studies, we found that call duration was independent of corresponding call intervals, and that it was negatively correlated with the corresponding inter-call interval. This indicates that echolocation call production mechanisms in the bat are highly correlated with the respiratory cycle. In contrast, call intervals in the mouse were directly correlated with call duration. Importantly, call duration was not, or was only slightly, correlated with inter-call intervals, consistent with the idea that vocal production in the mouse is largely independent of the respiratory cycle.

**Conclusions:**

Our findings suggest that ultrasonic vocalizations in mice are produced by call-pattern generating mechanisms that seem to be similar to those that have been found in primates. This is in contrast to the production mechanisms of ultrasonic echolocation calls in horseshoe bats. These results are particularly interesting, especially since mouse vocalizations have recently attracted increased attention as potential indicators for the degree of progression of several disease patterns in mouse models for neurodegenerative and neurodevelopmental disorders of humans.

## Background

Vocal communication is a complex behavioral pattern that occurs in most vertebrates. In most mammals, vocal output is produced by coordinated activity of several cranial, respiratory, and laryngeal muscles [1,2]. Most of these muscles are also involved in several other motor functions, such as swallowing, chewing, and respiration [3]. Hereby, the major challenge that has to be tackled by the vocal motor system is the coordination of vocal output with normal respiration [4].

**Figure 1:**
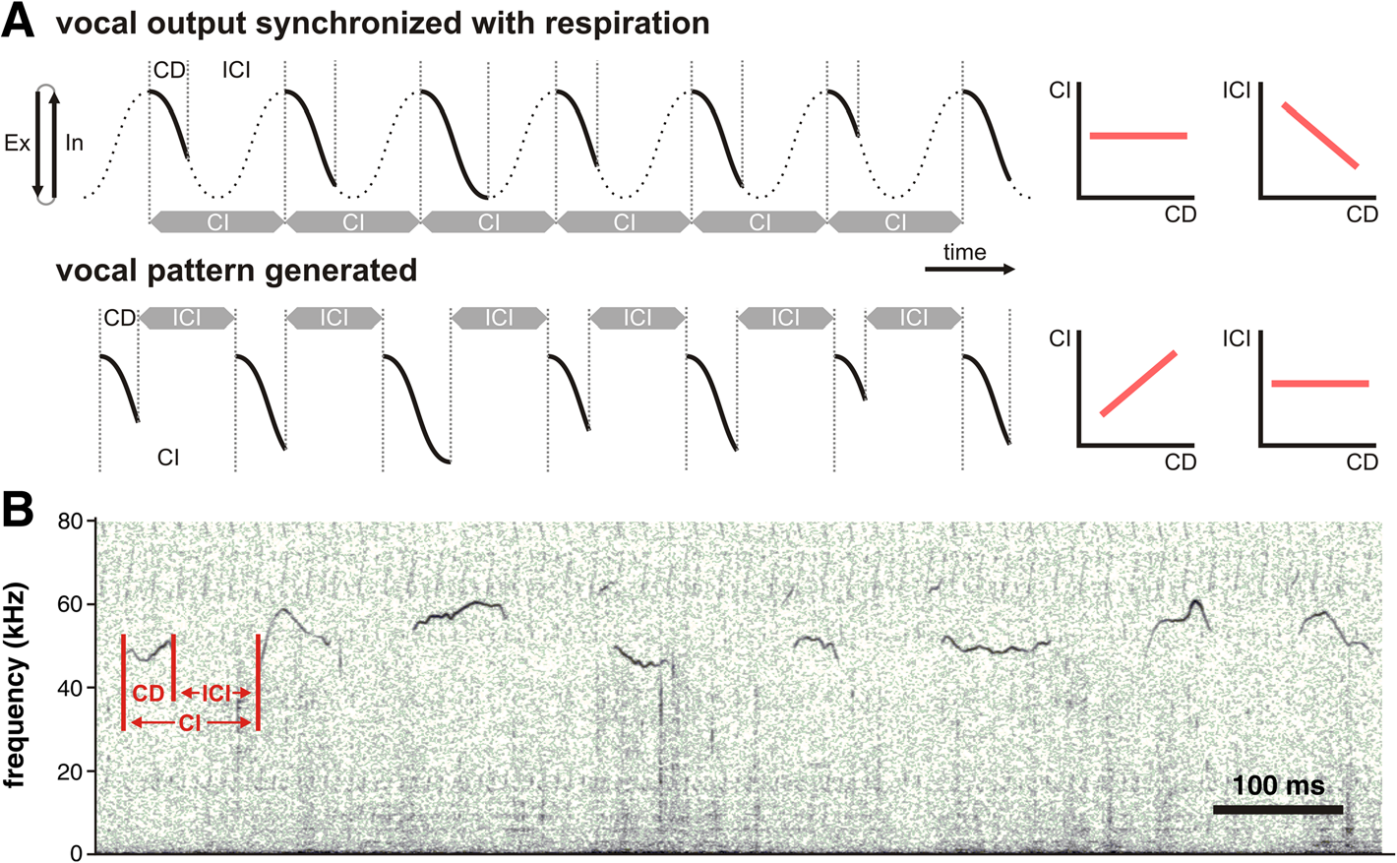
**Model of vocal production in mammals. (A)** Relationships between call patterns within two models of vocal production. (Top) Vocal output that is synchronized with the respiratory cycle and (bottom) vocalizations produced by a vocal pattern generator (see text for further explanations). Graphs with red lines on the right-hand side represent correlations between CD and CI as well as CD and ICI that result from each corresponding vocal production mechanism shown on the left hand side. Briefly, when synchronized to respiration, CI is determined by respiratory period and thus independent of CD. Moreover, the longer the CD, the shorter the ICI. On the other hand, with a pattern generator, CI will depend on CD whereas ICI will not. **(B)** Spectrogram of an example for several consecutive mouse vocalizations. *CD* call duration, *ICI* inter pulse interval, *CI* call interval.

One way to coordinate vocal utterances with respiration is to embed vocalizations into the expiratory phase of the normal respiratory cycle. This suggests that respiratory cycles are not, or are only slightly, modulated during vocal output, with the actual output recruiting an additional group of vocalization-related muscles in the lateral abdominal wall and the larynx [5-7]. Therefore, in this scheme, vocal behavior is characterized by a constant distance between call onsets of consecutive calls, so-called call intervals (CI). Moreover, CI is independent of the call duration (CD) within the expiratory phase, because CI is largely determined by the respiratory cycle. Additionally, in this scheme, CD is inversely proportional to the distance between the end of a call and the onset of the consecutive call [the so-called inter-call interval (ICI)], again because call onset is synchronized to respiration. These predictions are illustrated schematically in Figure 1A (top), in which we show a model of how vocalization characteristics would appear if vocal behavior was embedded into the respiratory cycle.

A vocalization mechanism embedded into the respiratory cycle can be found in a variety of animals, especially those producing vocal utterances with rapid succession. For example, bat species from the family Rhinolophidae [8,9], producing vocal sounds more than 25% of the time, usually produce one echolocation pulse per respiratory cycle at rest. These bats’ vocal onsets are highly correlated with expiratory onset [5,8,10], consistent with the abovementioned scheme of Figure 1A.

Another way to coordinate vocal output with respiration is to modulate the normal respiratory cycle by the vocal motor system. For example, vocalizations can be associated with phase shifts in respiratory rhythms or phase lags that are created by postponed inspirations [3,4,11,12]. In this case, CD has a significant effect on CI, because the latter is now largely determined by the vocalization itself instead of by an independent respiratory rhythm. Moreover, CD in this scheme would be only weakly correlated, if at all, with ICI. For example, in Figure 1A (bottom), a model of vocal behaviour that is independent of respiratory cycles is shown. As can be seen, in contrast to the model with vocalization embedded into the respiratory rhythm, this model shows very different patterns of predictions on the relationships between CD and CI or between CD and ICI.

Species that produce multi-syllabic vocal sequences, such as several primate species and humans, possess mechanisms to modulate respiration by vocalization, as in the scheme of Figure 1A (bottom). In these species, vocalizations are thought to be produced by vocal pattern generators within the brainstem [1,2,13]. However, these mechanisms are not fully understood, and this is even more so for inter-species differences. For example, while interactions between vocal and respiratory processes have been well studied in bats and primates, it is virtually unknown how these processes take place in other mammals, such as rodents. Mice produce genetically predetermined whistle-like ultrasonic vocalizations during several inter-individual encounters [14-16] (see Figure 1B for a sequence of mouse vocalizations). In this case, they produce their vocal utterances in very rapid succession [17], in a manner that is comparable to those of echolocation pulses in several bat species from the family Rhinolophidae [7,8,18]. This leads to the question of whether vocal utterances of mice are produced similarly to echolocation pulses in bats. In other words, are mouse vocal utterances embedded in the regular respiratory cycle with no or just minor adjustments of the respiration rate [5-7], or are they rather generated by a more complex mechanism possibly involving a vocal pattern generator that takes control over respiratory muscles [4,11]?

Studying vocal behaviour in mice is not only important for clarifying vocalization mechanisms in different species, but it can also have direct practical implications. Specifically, several mouse models have recently been established for understanding a broad spectrum of human disease conditions, including autism, speech disorders, and neurodegenerative diseases. Interestingly, vocal behavior is severely altered in these model animals and is therefore suggested to be a useful indicator for these impairments [19-22]. Before using mouse vocalizations as such indicators, however, we first have to investigate whether their production mechanisms, such as vocal pattern generation, are comparable to those in primates, including man, or not.

In the current study, we compared the mechanisms of ultrasound production in two different animal species, bats and mice, that both emit ultrasonic vocalizations in rapid succession, and in a comparable frequency range. Therefore, we analyzed the relationships between several call parameters such as CD, CI and ICI of mice and bats. Our data indicate that vocal production mechanisms of mice are different from those in bats and support the idea that they might be similar to those in primates.

## Results

**Figure 2:**
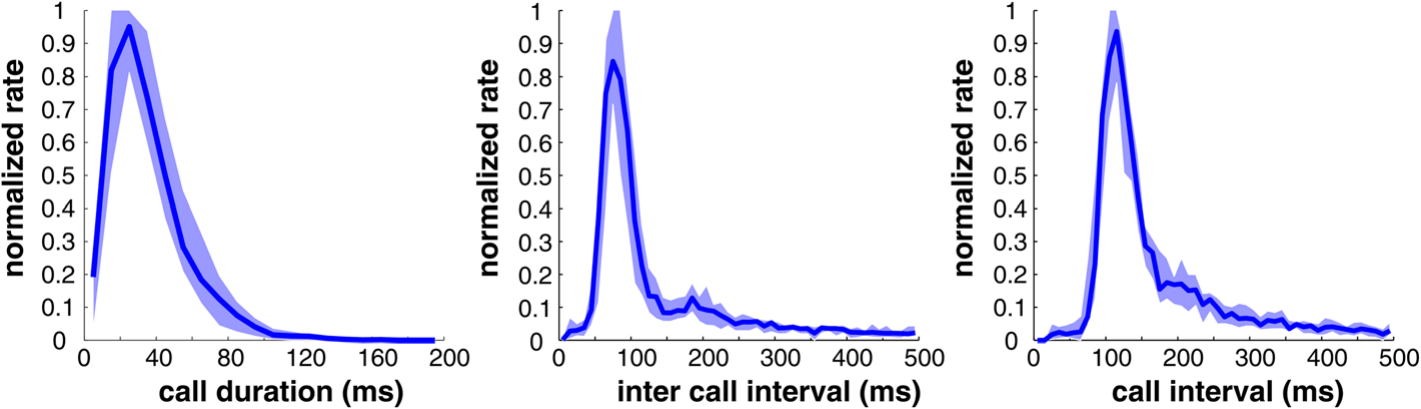
**Distribution of median call duration, inter-call interval, and call interval normalized for 18 mice.** Bin width 10 ms, shaded areas indicate 1st and 3rd quartiles.

**Figure 3:**
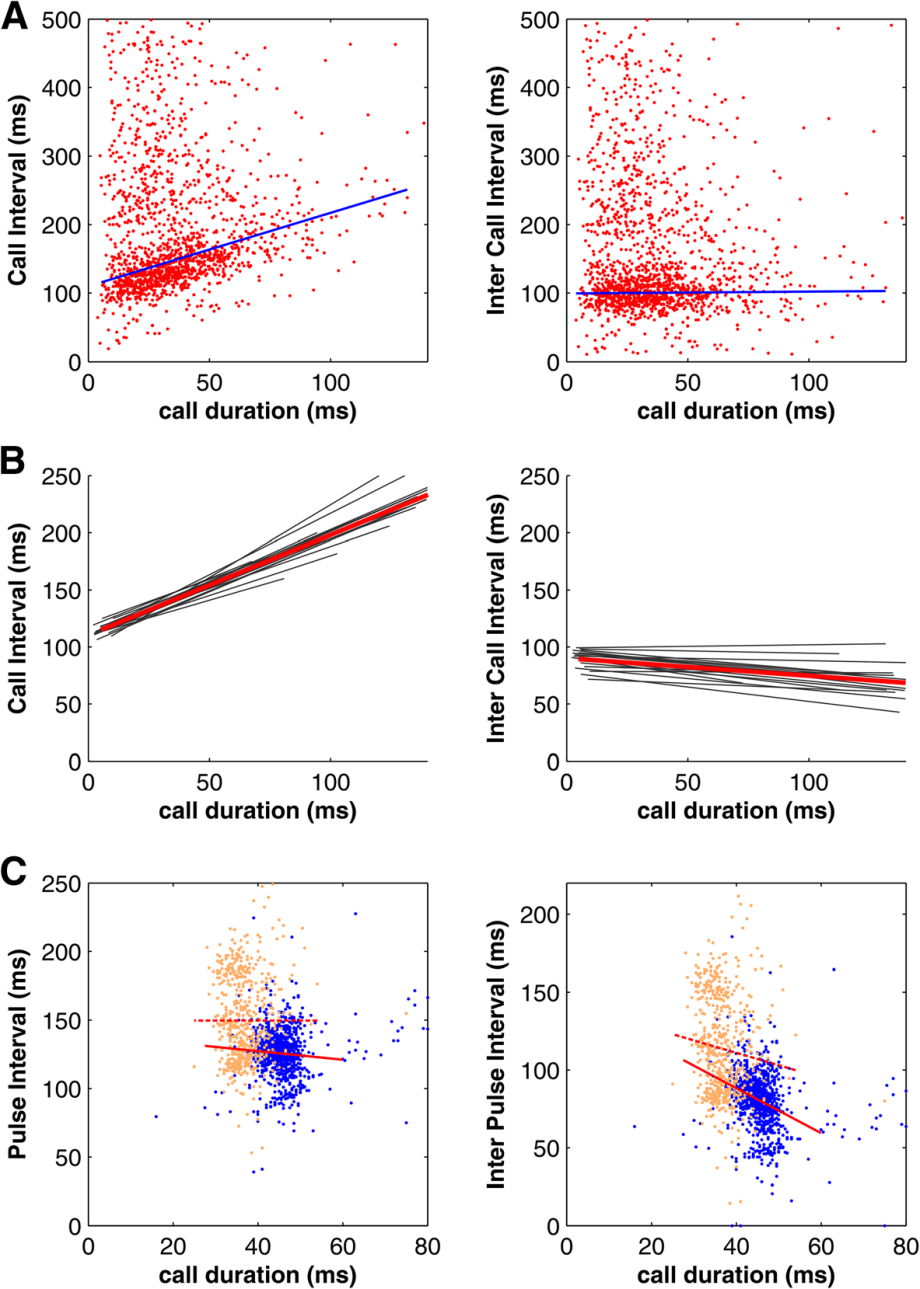
**Relationships between call parameters. (A)** Relationships between call parameters in an example mouse showing a significant correlation between CD and CI and no correlation between CD and ICI. **(B)** Regression lines for the relationships of call parameters for all mice showing a significant stronger correlation between CDs and CI that between CD and ICI. Red lines indicate the mean regression lines **(C)** Relationships between call parameters in two horseshoe bats (a different color is used for each bat individually) indicating no correlation between CD and CI, but a significant negative correlation between CD and ICI. Dashed and solid lines indicate regression lines for the relationships between call parameters of bat #1 and #2, respectively.

We recorded 50,797 vocalizations from 18 house mice (2,822 ± 702 (SEM) per mouse). Median CD calculated over all 18 mice was 33 ms (see Figure 2). ICI and CI occurred with highest probability between 50 to 100 ms and 90 to 140 ms, respectively, in all mice (see Figure 2). Figure 3A shows the relationship between call parameters collected from a single representative mouse. As can be seen from the figure, CD was significantly correlated with CI (correlation coefficient: 0.60, p < 0.0001, Pearson’s correlation), and the data showed a steep slope of the regression line of 1.08. In contrast, CD was not correlated with ICI (correlation coefficient: 0.03, p > 0.1), and the data showed a flat slope of the regression line of 0.03. We performed similar analyses for each mouse individually and found that CD was significantly correlated with CI for every single animal (mean correlation coefficient 0.46 ± 0.02 and mean regression slope 0.88 ± 0.03; see Figure 3B and Table 1). In contrast, CD was significantly correlated with ICI in only 11 mice, and in those animals, the overall correlation coefficients were low (mean correlation coefficient −0.15 ± 0.02). In fact, the regression lines showed flat or weakly decreasing (negative slopes) in all mice (mean slope −0.15 ± 0.02; see Figure 3B and Table 1). Finally, we tested whether CD shows a stronger correlation to CI or ICI and found that slopes of regression lines were significantly steeper when correlating CD with CI than CD with ICI (p < 0.01, Wilcoxon signed rank test). Thus, mouse vocalizations showed a pattern of results consistent with the scheme of Figure 1A (bottom).

**Table 1:** Overview of the Pearson’s correlation and regression analyses made for every single mouse individually

***Mouse***	***CD versus CI***			***CD versus ICI***		
	***p-value***	***corr. coef***	***regr. slope.***	***p-value***	***corr. coef***	***regr. slope.***
*1*	**<0.0001**	0.60	0.92	**0.0004**	−0.05	−0.04
*2*	**<0.0001**	0.53	0.91	**<0.0001**	−0.25	−0.25
*3*	**<0.0001**	0.58	0.86	**<0.0001**	−0.21	−0.17
*4*	**<0.0001**	0.47	0.89	**<0.0001**	−0.24	−0.21
*5*	**<0.0001**	0.60	1.08	0.400	0.03	0.03
*6*	**<0.0001**	0.39	0.85	0.465	−0.04	−0.04
*7*	**<0.0001**	0.38	0.70	**0.0008**	−0.18	−0.17
*8*	**<0.0001**	0.40	0.74	0.022	−0.16	−0.16
*9*	**<0.0001**	0.50	0.96	0.050	−0.18	−0.18
*10*	**<0.0001**	0.32	0.62	0.022	−0.20	−0.21
*11*	**0.003**	0.33	0.85	0.050	−0.20	−0.32
*12*	**<0.0001**	0.51	0.90	**<0.0001**	−0.26	−0.20
*13*	**<0.0001**	0.45	0.89	**<0.0001**	−0.24	−0.26
*14*	**<0.0001**	0.52	0.92	0.047	−0.08	−0.09
*15*	**<0.0001**	0.25	0.77	**0.0004**	−0.10	−0.19
*16*	**<0.0001**	0.46	0.89	**<0.0001**	−0.22	−0.25
*17*	**<0.0001**	0.43	0.78	0.388	−0.06	−0.06
*18*	**<0.0001**	0.63	1.28	0.887	−0.01	−0.01

Data in boldface: p < 0.01.

The mouse results above were categorically different from those obtained with bats. Specifically, to obtain control data for the characteristics of mouse vocal utterances obtain above, we measured such utterances from a model animal in which vocalization is known to be synchronized with the respiratory cycle. We characterized CD, CI, and ICI from 1,532 echolocation pulses of two horseshoe bats (see Figure 3C). Both animals showed no correlation between CI and CD (correlation coefficients: -0.00 and 0.07, p > 0.1 and p > 0.05, Pearson’s correlation) with flat slopes of the regression line of −0.00 and −0.31, respectively. ICI were significantly negatively correlated with CD (correlation coefficients: -0.10 and −0.29, p < 0.05 and p < 0.0001) and showed steep slopes of the regression line of −0.80 and −1.44, respectively. Thus, the echolocation patterns from bats, with known synchronization to respiratory rhythms, were qualitatively different from those of mice, supporting the hypothesis that the latter possess different vocalization mechanisms.

## Discussion

We analyzed the distribution of CD, CI, and ICI in mouse vocalizations and compared them to the echolocation calls of bats. Our results suggest that – in contrast to echolocation calls in bats – mouse vocalizations might be produced by similar pattern generating mechanisms to those found in primates.

In echolocation calls of both bats, CD was independent of CI but negatively correlated with ICI. As explained in Figure 1A (top), this indicates that vocal production mechanisms in echolocating horseshoe bats are highly correlated with the respiratory cycle. These results are in accordance with previous studies on echolocation calls in horseshoe bats [5-8] indicating that these two bats in our study were representative of the larger bat population. Our findings, therefore, confirm the hypothesis that echolocation pulses are embedded within the respiratory cycle as has been supposed for animals producing vocal output with rapid succession [10]. In contrast, we observed a direct correlation between CI and CD in house mice; CD, however, was not, or only slightly, correlated with ICI. These findings indicate that vocal production in mice is largely independent from the respiratory cycle, and our results therefore point to a vocal production mechanism that modulates the normal respiratory cycle. Such modulation of the respiratory cycle has been observed during vocal behavior in primates including man [3,4,11,12]. Here, distinct vocal utterances are produced by pattern generating networks in the brainstem that control several vocalization-related cranial and abdominal muscles [13,14,23]. These, in turn, modulate the normal respiratory cycle, either by shifting the phase of respiratory rhythms or by generating phase lags that are created by postponed inspirations [4,12]. Here, it is important to mention that horseshoe bats possess a rich repertoire of non-echolocation social calls in addition to their echolocation calls [24]. Further studies will have to investigate whether or not the non-echolocation social calls of bats might have a vocal pattern generating mechanism more similar to mice than their echolocation calls.

Despite the fact that mice produce their ultrasonic vocal utterances with rapid succession, our results suggest that vocal production mechanisms in mice are rather independent from the normal respiratory cycle, similarly to those found in primates. This is of particular interest, since mouse vocalizations are increasingly being used as indicators for the degree of progression of several disease patterns in mouse models for neurodegenerative and neurodevelopmental disorders of humans (e.g. [19-22,25]). In these models, it seems that mouse vocalizations are altered similarly as human speech in the corresponding neurodegenerative and neurodevelopmental disorders. At first glance, these findings may seem very surprising in light of the different production mechanisms for human speech and vocal utterances of non-human primates and mice. Learned vocal patterns, such as human speech are produced primarily by a complex network of cortical areas. In contrast, genetically pre-programmed vocalizations of non-human primates are generated by a complex neuronal network in the brainstem [1,2,14,26]. However, recent work is revealing that such differences are not as dichotomous as one might think. For example, several studies indicate a two-stage model for the evolution of human speech, a model that is based on the integration of primate-general brainstem mechanisms of acoustic communication with human-specific motor capacities to produce articulate speech (for review [26]). Therefore, due to similar mechanisms at least at the level of the primate-general brainstem mechanisms, our findings support the idea of using mouse vocalizations as an animal model for vocal motor control mechanisms.

## Conclusion

During vocal behavior in mice, CI and CD are directly correlated, while CD is not, or is only slightly, correlated with ICI. These findings are in contrast to echolocation call pattern distributions in bats and indicate that vocalizations in mice are likely produced by a call-pattern generating mechanism that is capable of modulating respiration in these animals. Thus, these results suggest a similar call pattern generating mechanism in mice and in primates, including man, and support the idea of using vocal behavior in mice as a potential marker for the degree of progression of several disease patterns in mouse models for neurodegenerative and neurodevelopmental disorders in humans.

## Methods

### Animals

In the present study, we used 18 sexually mature male mice (10 to 24 weeks of age, *Mus musculus*, NMRI strain) and 2 male Greater Horseshoe Bats, *Rhinolophus ferrumequinum*. The experiments on mice were performed at the University of Tübingen, Germany, and those on bats at UCLA, U.S.A. Procedures were in accordance with NIH guidelines for experiments involving vertebrate animals and were approved by UCLA’s Animal Research Committee, Los Angeles, USA, and the Regierungspräsidium Tübingen, Germany.

### Data acquisition and analysis

Acoustic recordings were made in sound-attenuated chambers for both species. Mice vocalizations were recorded during male–female encounters. Male mice were stimulated to increase vocal output by placing a female mouse in a small wire box in the male’s home cage. This approach ensured restricted tactile and olfactory contact between the mice and avoided copulation, which is known to abort vocal output in male mice. Emitted vocalizations of the mice were captured by a custom-made ultrasound condenser microphone (University of Tübingen) placed 10 to 15 cm (depending on the mouse’s body position) above the head of the mouse. Bats were mildly restrained in a foam sandwich while their heads remaining mobile for acoustical stimulation. Bats emitted echolocation calls spontaneously. We acoustically stimulated the animals with playbacks of their own calls to increase call performance. Echo playbacks (echo mimics) were generated as described previously (e.g., [27,28]). Briefly, echolocation calls were captured by a ¼-inch microphone (4939 with preamplifier 2633, Brüel & Kjær, Nærum, Denmark) positioned 15 cm ahead of the bat’s head, and these calls were then played back with a 4 ms-delay (produced electronically with Tucker-Davis Technology (TDT) system III hardware and the openEX software) though an ultrasonic loudspeaker placed approximately 20° laterally and 10 cm in front of the bat’s left ear.

During the recording of the mice vocalizations, the acoustic signal was digitized with an A/D converter (sample rate 256 kHz; PCTape, University of Tübingen) and stored on a notebook. Custom-made software (Selena®, University of Tübingen) was used to manually detect call on- and offsets. For bat calls, acoustic signals were digitized with a CED Mikro1401 mk II system (sample rate 200 kHz; Cambridge Electronic Design, Cambridge, UK) and recorded with Spike 2 software. In both cases, custom-made software (MATLAB, Mathworks) was then used to calculate CD, ICI and CI. CD was defined as the time between call onset and offset; CI as the time between the onsets of two consecutive calls; and ICI as the time between the end of a call and the beginning of the following vocalization (see Figure 1B for a sequence of mouse vocalizations).

### Statistical analysis

Statistical analysis was performed with MATLAB (Mathworks Statistics Toolbox). We used the Pearson’s correlation (p < 0.01) to examine the correlation between CD and ICI as well as between CD and CI. A Wilcoxon signed rank test (p < 0.05) was performed to test for significant differences in slopes of regression lines between both correlations tested.

## Abbreviations

CD: Call duration; CI: Call interval; ICI: Inter-call interval.

## Competing interests

None of the authors have any competing interests.

## Authors’ contributions

SRH designed the study, interpreted the data, and wrote the manuscript; NG, SF, and AMS performed the experiments; SRH, NG, SF, and AMS analyzed the data. All authors read and approved the final manuscript.
